# Difference in the Care of Patients with Amyotrophic Lateral Sclerosis With and Without Intervention from the Palliative Care Team: Observations from a Center in Japan

**DOI:** 10.1089/pmr.2020.0084

**Published:** 2021-07-02

**Authors:** Kazuya Takahashi, Fumi Murakami, Kiyonobu Komai, Chiho Ishida, Yuko Kato-Motozaki

**Affiliations:** ^1^ Department of Neurology, Hokuriku Brain and Neuromuscular Disease Center, National Hospital Organization Iou National Hospital, Kanazawa, Japan.; ^2^ Palliative Care Team, Hokuriku Brain and Neuromuscular Disease Center, National Hospital Organization Iou National Hospital, Kanazawa, Japan.; ^3^ Nursing Unit, Hokuriku Brain and Neuromuscular Disease Center, National Hospital Organization Iou National Hospital, Kanazawa, Japan.

**Keywords:** amyotrophic lateral sclerosis, opioid, palliative care

## Abstract

***Background:*** Despite the significant palliative care needs for people living with amyotrophic lateral sclerosis (ALS), palliative medicine in Japan is mainly focused on oncologic disease.

***Objective:*** To compare the care provided to patients with ALS with and without intervention from the palliative care team (PCT and non-PCT groups, respectively).

***Design:*** This is a retrospective case–control study.

***Setting:*** One ALS center in Japan.

***Participants:*** Sixty patients with clinically definite ALS treated until death from January 2012 to December 2019.

***Measurements:*** We compared the two groups based on the presence of advance directives, patient age, use of noninvasive and invasive ventilation, maximum opioid dosage, and use of nonopioid palliative medications such as antidepressants and anxiolytics. We also compared the prescribing practices of the attending physicians.

***Results:*** There was no difference in the rate of advance directive completion between the PCT and non-PCT groups. Although all but one patient in the PCT group used opioids, only half of the patients in the non-PCT group used opioids (*p* < 0.001). The mean maximum opioid dosage was higher in the PCT group than in the non-PCT group (*p* = 0.003). Moreover, 79.2% and 41.7% of the PCT and non-PCT groups, respectively, received antidepressants or antianxiety agents (*p* = 0.004). Maximum opioid dosages were not different on the basis of attending physician's experience level.

***Conclusions:*** Opioid and nonopioid medications intended for symptom management were more likely to be prescribed to patients with ALS who received intervention from a PCT.

## Introduction

Amyotrophic lateral sclerosis (ALS) is a neurodegenerative disease characterized by loss of motor function that almost inevitably leads to death due to respiratory failure.^1^ The annual incidence is ∼1.5–2.5 per 100,000 population and the mean survival of patients with ALS without mechanical ventilation is two to four years.^2,3^ Although the importance of palliative medicine in neurodegenerative diseases such as ALS is newly being realized worldwide,^4^ in Japan, the specialty is still mainly focused on oncology. The Japanese Society for Palliative Medicine (JSPM) registers palliative care teams (PCTs) every year, with 520 registered in 2018.^5^ In 2018, a total of 97,162 patients, including both outpatients and inpatients, received intervention from PCTs, 4.5% of which were for patients without cancer, and only 8.7% of interventions for patients without cancer were for neurological diseases. It is important to note that specific details such as the number of staff, occupation, and number of interventions are limited because the data are from a summary report from each hospital.^5^

Multidisciplinary clinics are globally being recognized as the most ideal way to provide care to patients with ALS.^6,7^ However, incorporating palliative care specialists into multidisciplinary ALS teams is still in the nascent stage.^8^ In Japan, palliative care physicians are certified by the JSPM and palliative care nurses (the Certified Nurses for Palliative Care [CNPC]) are certified by the Japanese Nursing Association. As of 2020, there were only 273 fellows of JSPM^9^ and 2438 CNPCs registered in Japan.^10^ There are not enough specialists to cover a country of >120 million people. Furthermore, most hospitals other than cancer centers do not have a dedicated PCT. The JSPM fellows and CNPCs belong to other divisions such as internal medicine and their work is focused there rather on palliative care. As such, palliative care interventions are typically only initiated upon request from the primary medical team and are historically focused on oncology patients. Patients with neurodegenerative diseases could likely benefit greatly from palliative care intervention, but the current structure of palliative medicine in Japan means that even patients with intractable neurodegenerative diseases do not receive sufficient palliative care.

Neurologists have few opportunities for formal training of palliative medicine skills such as opioid prescribing, and knowledge depends on personal experience. This lack of consistent training means there is a significant variety in symptom management of seriously ill patients, including those living with ALS.

ALS treatment guidelines in Japan^11^ recommend that patients with ALS have multidisciplinary care, but palliative care is not specifically mentioned as a component of this. Since the impact of multidisciplinary PCT interventions on the care of patients with ALS is still unclear in Japan, in this study, we examined the differences in the care provided to patients with ALS with or without intervention from the PCT (PCT group and non-PCT group, respectively) at a center specializing in intractable medicine. We compared the PCT and non-PCT groups with regard to the presence of advance directives, patient age, frequency of noninvasive or invasive ventilation use, maximum dosage of opioids, and the use of nonopioids such as antidepressants and antianxiety agents, which are frequently used in palliative care. We also analyzed the prescribing patterns of attending physicians and the rate of PCT consultation for their patients with ALS. Our hypothesis was that patients with ALS who did not receive PCT intervention would have lower rates of palliative interventions, including opioid use and advance directive completion.

## Materials and Methods

### Japanese medical system and the specialty of the Iou National Hospital

In Japan, the Ministry of Health, Labour and Welfare divided Japanese hospitals into two hospital types: psychiatric and general. General hospitals are further divided into two types: those with general beds and those with long-term care beds.

Intractable diseases such as ALS are the designated focus of some national hospitals for research and treatment. These hospitals specializing in intractable diseases have long-term care beds for patients with such diseases. Iou National Hospital is a national hospital specializing in the field of intractable neuromuscular diseases and is the only center with long-term care beds for these patients in the Hokuriku area of Japan.

### Activity of the PCTs in Iou National Hospital

The PCT of Iou National Hospital consists of three physicians (two neurologists and a pediatrician), a CNPC, a certified nurse for chronic respiratory nursing, an outpatient nurse, seven ward nurses, three certified public psychologists, a pharmacist, a physical therapist, an occupational therapist, a speech therapist, a medical social worker, and a registered dietitian. The PCT initiates intervention only upon request from the primary medical team involved with the inpatients or outpatients. The PCT functions as a consulting service for the patients and participates in a monthly meeting of the entire care team to discuss each patient. They offer recommendations that are at the discretion of the primary neurologist to put into action. The PCT continues to follow each patient until the care team determines that no further interventions are needed. In addition to a monthly meeting, patient-specific meetings are also a major activity for the PCT. In brief, the primary medical team raises issues such as controlling anxiety with the PCT team. The PCT members then provide recommendations to the primary team for management of these issues based on prognosis, advance directives, discussions with patient and family, and chart review. These patient-specific meetings are generally repeated every two to six weeks to evaluate the current interventions and plan the next intervention.

### Patient selection

A group of patients who were diagnosed with clinically definite (revised El Escorial criteria) or pathologically definite ALS, and treated until death at Iou National Hospital from January 2012 to December 2019, formed the sample. After excluding 6 patients because of their vegetative state due to anoxic encephalopathy caused by suffocation due to aspiration or sputum clogging (2 patients), complications of malignant lymphoma with central nervous system infiltration (one patient), or a lack of detailed information (3 patients), the data of 60 patients with ALS were analyzed in this study.

Patients who had regular interventions from the multidisciplinary PCT—as explained above—were designated as the PCT group and those who did not have intervention with PCT were designated as the non-PCT group. Among them, 24 patients were classified into the PCT group, whereas 36 patients were categorized into the non-PCT group. The study was approved by the hospital's ethics committee (2018-1).

### Data collection

We retrospectively obtained the following information from the electronic medical record system: the attending physician's name, disease duration (period from the date of onset of initial symptoms to the time of death), the presence of advance directives (preferences regarding feeding tube placement, noninvasive positive pressure ventilation, tracheostomy, and code status), patient age, frequency of noninvasive or invasive ventilation use, maximum dosage of opioids prescribed, and the use of nonopioids such as antidepressants and antianxiety agents, which are frequently used in palliative care. In addition, we calculated the PCT intervention rate (the number of patients with ALS with PCT intervention/the number of all patients with ALS being treated by each attending physician) × 100 (%).

### Calculation of maximum opioid dosage

For most patients with ALS in this study, opioids were used for shortness of breath. The maximum daily opioid dosage stated in the regular outpatient prescription or inpatient prescription until death was taken as the maximum opioid usage. If rescue use of opioids was required irregularly, such as once every few days, the opioid dosage for rescue use was excluded from the daily opioid dosage. In Japan, only morphine preparations are allowed for patients with intractable diseases other than cancer. Nevertheless, since fentanyl was used in two patients with ALS in this study, 2.1 mg/3 days of fentanyl was calculated to be equivalent to 30 mg/day of morphine hydrochloride.

### Statistical analysis

The unpaired Student's *t*-test and Mann–Whitney *U*-test were used to compare groups based on whether the data demonstrated normality. The Wilcoxon signed-rank test was used for comparing two paired datasets. Upon comparing the contingency table, in case of five or more in all cells, Pearson's chi-square test was used; otherwise, Fisher's exact test was used. *p*-Values <0.05 were considered significant. All quantitative data were presented as mean (standard deviation). Statistical analyses were performed using PASW Statistics for Windows, Version18.0. Chicago: SPSS, Inc.

## Results

### Demographics of the PCT and non-PCT groups

Clinical characteristics for the two groups are given in Table 1. Disease duration and rates of advance directive completion and mechanical ventilation use were similar between the two groups. The age at death in the PCT group was significantly lower than that in the non-PCT group. The PCT group had a significantly higher rate of opioid usage than the non-PCT group (95.8% vs. 50.0%, *p* < 0.001) and usage of antidepressants or anxiolytics was higher in the PCT group as well (79.2% vs. 41.7%, *p* = 0.004).

**Table 1.** Sample's Clinical Characteristics

	PCT	Non-PCT	*p*
Male/female	12/12	18/18	
Age at death (years old [SD])	66.4 (9.98)	73.1 (10.2)	0.014^a^
Mean disease duration (months [SD])	41.4 (31.4)	51.6 (36.5)	0.334^b^
Existence of advance directives	22/24 = 91.7%	29/36 = 80.6%	0.299^c^
No. of patients with ventilator management	12/24 = 50%	14/36 = 38.9%	0.395^c^
TPPV	1/24 = 4.2%	6/36 = 16.7%	0.225^d^
No. of patients receiving opioids	23/24 = 95.8%	18/36 = 50%	<0.001^c^
Mean maximum opioid dosage (mg/day [SD] equivalent to morphine hydrochloride)	70.3 (76.0)	27.0 (22.7)	0.003^b^
No. of patients receiving nonopioid drugs such as antidepressants or antianxiety agents	19/24 = 79.2%	15/36 = 41.7%	0.004^c^

^a^ Unpaired Student's *t*-test.

^b^ Mann–Whitney *U*-test;

^c^ Pearson's chi-square test.

^d^ Fisher's exact test.

PCT, patients with amyotrophic lateral sclerosis with intervention from the palliative care team; non-PCT, patients with amyotrophic lateral sclerosis without intervention from the palliative care team; TPPV, tracheostomy positive pressure ventilation.

### Trend of opioid use in patients with ALS

As palliative care has been included in Japanese ALS treatment guidelines^6^ and considering the gradual increase in the frequency of opioid use in patients with ALS,^12^ we next compared data from 2012 to 2015 with data from 2016 to 2019 to observe any changes in opioid prescribing over time. Opioid usage increased over time: 37.5% of the non-PCT patients who died between 2012 and 2015 used opioids as opposed to 60.0% of the patients who died between 2016 and 2019 (*p* = 0.040).

**FIG. 1.**
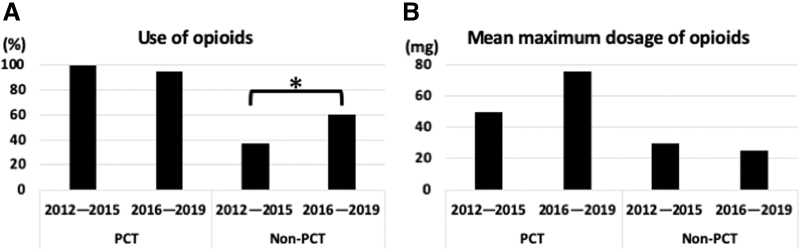
Trend of opioid use in patients with ALS. The bar graphs depict the trend in the proportion of patients receiving opioids **(A)** and the mean maximum dosages **(B)** in the PCT and non-PCT groups. Based on the period of death, both groups are segregated into two periods: 2012–2015 and 2016–2019. *p = 0.040 (Pearson's chi-square test). ALS, amyotrophic lateral sclerosis; PCT, palliative care intervention group; non-PCT, nonpalliative care intervention group.

The mean maximum opioid dosage was not different between the non-PCT patients who died between 2012 and 2015 (30.0 [15.8] mg/day of morphine hydrochloride) and those who died between 2016 and 2019 (25.5 [26.0] mg/day of morphine hydrochloride) (*p* = 0.119). However, in the PCT group, the mean maximum opioid dosage tended to increase to 79.4 (83.9) mg/day of morphine hydrochloride in patients who died between 2016 and 2019 from 44.7 (42.7) mg/day of morphine hydrochloride in those who died between 2012 and 2015 (Fig. 1B) (*p* = 0.233).

### Differences in opioid use by the prescribing physician

Since the PCT intervention is only initiated upon request from the primary medical team and PCT recommendations are only implemented by the choice of the attending physician, there is the possibility of significant differences in PCT consultation and prescribing practices between the 14 different neurologists who were responsible for the patients included in this study. Three of the physicians never requested PCT intervention, 1 only had 1 patient with ALS and requested consultation, and the remaining 10 had variable rates of PCT intervention consultation. The mean of maximum opioid dosage used by each physician was higher in PCT patients than in non-PCT patients (*p* = 0.037). There was no difference in opioid dosage between physicians with >10 years of clinical experience and those with <10 years of clinical experience (*p* = 0.477). Between the two time points, most attending physicians increased the rate of PCT consultation for their patients with ALS.

## Discussion

Overall, patients with ALS who received PCT intervention were more likely to be prescribed opioid and nonopioid palliative medication than those who did not receive PCT intervention. Other interesting differences between the two groups were found as well. For example, the age at death of patients in the non-PCT group was significantly higher than that in the PCT group. This may have been due to the difference in the number of patients who underwent a tracheostomy between groups (one in the PCT group, six in the non-PCT group), which is thought to extend life. With the exclusion of the patients who underwent a tracheostomy, the mean age at death did not significantly differ between the PCT group (66.8 [9.95] months, range 39–81) and the non-PCT group (72.2 [10.32] months, range 43–86) (*p* = 0.058). Another factor that could contribute to this difference is the tendency for younger patients to receive palliative care consults due to higher rates of anxiety and distress. In general, younger patients are less likely to accept their illness and death than older patients. Moreover, anxiety about their future life may be more prevalent when their family is also young. These anxieties may have triggered the PCT intervention in younger patients with ALS.

Different rates of tracheostomy positive pressure ventilation (TPPV) between groups may have also contributed to the mean maximum opioid dosage difference. Owing to the difficulty in communicating with patients with ALS who have TPPV, it may be difficult for care staff to effectively evaluate their dyspnea.^13,14^ This may reduce the chances of including opioids in palliative care for patients with TPPV. Indeed, in patients with ALS who have TPPV, opioids were prescribed in only one patient in the PCT group. However, a significant difference in the maximum opioid dosage between in the PCT group and the non-PCT group remained even if the data of the patients with TPPV were omitted (*p* = 0.003).

Knowledge of palliative medicine in Japan is also an important issue. In a 2010 study, Ogino reported that ∼21% of the fellows of the Japanese Society of Neurology prescribed morphine, but 77% of them had experience with five or fewer patients.^12^ Since all of the physicians in this study had experience with opioid prescribing for their patients, it is likely that these neurologists have more palliative care knowledge and experience than more general neurologists. Furthermore, in this study, 13 physicians were involved in the care of 36 patients with ALS without intervention from the PCT and 10 of them had experience of using opioids in the care of some patients with ALS along with the PCT. Since the frequency of opioid use in patients with ALS increased between 2016 and 2019 compared with that between 2012 and 2015, it is possible that physicians were learning PCT skills and becoming more comfortable with opioid prescription for ALS by this collaboration, although the dosage of opioids might still be inadequate.

In addition, in some patients from the 2016–2019 cohort, PCT intervention was initially suggested by a nonphysician member of the primary team. This suggests that other team members recognized the benefit of having the PCT involved in patient care. Team members such as nurses may be able to more accurately assess dyspnea in patients with ALS and thus should contribute to deciding when the PCT should be involved. Education for nonmedical palliative care providers such as caregivers, the patient's family, and the patient themselves may be even more useful for the proper initiation of PCT intervention. In addition, sharing online medical records may facilitate multidisciplinary PCT intervention in the attending clinics.

### Limitations

Since this study is based on a small sample of patients from a single center in one region of Japan, it does not show trends throughout Japan. Owing to small sample size, the differences in which PCT members saw each patient could affect the results, as the members of the PCT are all of widely varying experience and knowledge of palliative care. Furthermore, the clinical form of ALS can also have a significant impact on the content of palliative care, and differences in the difficulty in communicating with each patient with ALS may significantly change the understanding of their dyspnea. To minimize these effects, it is necessary to conduct further studies at multiple facilities, including many areas in Japan with a larger number of patients.

## Conclusion

This study showed that PCT intervention was associated with higher rates of opioid use and higher average doses of opioids in patients with ALS. It also showed that the neurologists became more likely to request PCT intervention over time. It is possible that this increase in collaboration also helps educate nonpalliative care primary clinicians on palliative medicine skills such as appropriate opioid prescription for dyspnea due to respiratory failure in ALS.

## Abbreviations Used

ALS: amyotrophic lateral sclerosis
CNPC: Certified Nurses for Palliative Care
PCT: palliative care team
JSPM: Japanese Society for Palliative Medicine
TPPV: tracheostomy positive pressure ventilation
